# Preservation of Health

**Published:** 1888-01

**Authors:** 


### PRESERVATION OF HEALTH.

It is impossible to lay down any rules for health which may be followed safely by all persons. Health depends largely upon the diet. Some people cannot eat newly baked bread; others cannot eat it when stale. Much fresh meat, with some constitutions, induces fullness of the head and a feverish state of the system, because it makes blood too fast.

It should therefore be discarded, and a little salt meat or fish, if the appetite craves it, with fresh fruit and vegetables, will be found, probably, to be just what the system requires. In truth, with health, as in many other things, each person must be a law unto himself. In acute or intricate cases, physicians are necessary, but in many minor matters they cannot decide. It is true that what is "one man's meat may be another's poison," and a little poisoning, now and then, seems indispensable to teach us our individual physical, as well as mental idiosyncrasies.

Experience thus gained, if not carried to such an excess as to prove too severe a schoolmaster, will be of more value through life than all the doctors in Christendom—with all respect be it spoken—besides saving many a long bill at the drug store.

Children should be taught at an early period in life to avoid the use of condiments Their food should be plentiful, but simple. Many a mother will give her very young children rich food—pastry, cake and sauces and condiments of the most indigestible or fiery kind—and tell you her children are healthy, and nothing hurts them. Perhaps the injury is not apparent at first, but it will not be long before headaches, indigestion of the most serious character, dyspepsia, fixed for life, disproves the truth of her opinions.

